# Cross-cultural adaptation and construct validity of the German version of the Adult Social Care Outcomes Toolkit for service users (German ASCOT)

**DOI:** 10.1186/s12955-020-01533-7

**Published:** 2020-10-06

**Authors:** Birgit Trukeschitz, Judith Litschauer, Assma Hajji, Judith Kieninger, Adiam Schoch, Juliette Malley, Stacey Rand, Ismo Linnosmaa, Julien Forder

**Affiliations:** 1 Research Institute for Economics of Aging, WU Vienna University of Economics and Business, Welthandelsplatz 1, 1020 Vienna, Austria; 2 Care Policy and Evaluation Centre, London School of Economics and Political Science, London, UK; 3 Personal Social Services Research Unit, University of Kent, Canterbury, UK; 4 Centre for Health and Social Economics, Finnish Institute for Health and Welfare, Helsinki, Finland; 5 Department of Health and Social Management, University of Eastern Finland, Kuopio, Finland

## Abstract

**Background:**

There has been considerable interest in using the Adult Social Care Outcomes Toolkit (ASCOT), developed in England, to measure quality-of-life outcomes of long-term care (LTC-QoL) service provision in national and cross-national studies.

**Objectives:**

The aim of this study was to translate and culturally adapt the original ASCOT service user measure into German and to evaluate its content and construct validity in Austrian home care service users.

**Methods:**

The translation and cultural adaptation process followed the ISPOR TCA guidelines. We used qualitative data from six cognitive debriefing interviews with Austrian recipients of home care services to assess linguistic and content validity. In addition, cross-sectional survey data (*n* = 633) were used to evaluate construct validity by testing hypothesized associations established in a previous study for the original English ASCOT service user instrument.

**Results:**

Cognitive debriefing interviews confirmed that the German adaptation of the ASCOT service user instrument was understood as intended, although two domains (‘Control over daily life’ and ‘Dignity’) and selected phrases of the response options were challenging to translate into German. All ASCOT domains were statistically significantly associated with related constructs and sensitive to service user sub-group differences.

**Conclusions:**

We found good evidence for a valid cross-cultural adaptation of the German version of ASCOT for service users. The analysis also supports the construct validity of the translated instrument and its use in evaluations of QoL-effects of LTC service provision in German-speaking countries. Further research on the reliability and feasibility in different care settings is encouraged.

## Background

Long-term care (LTC) and health care spending are expected to be the main drivers of age-related expenditures in Europe for the next decades [1]. Policy makers, care managers and other stakeholders in this field, however, are not only interested in the development of LTC costs but are also keen to learn more about the impact of public spending for LTC benefits and services on people’s lives. Policy and practice prepare responses to demographic and societal changes which affect both supply of and demand for long-term care. Thus, tools are needed to improve the evidence base of the effectiveness of LTC services to better inform LTC reforms.

Measuring the effects of LTC services on the quality of life (QoL) of LTC recipients requires instruments that both capture the LTC service impact on relevant areas of life sufficiently well and are available in the language of the country of interest. Well-known QoL-measures available in German comprise health-related quality of life (HRQoL), such as EQ-5D [2], or ICECAP-O [3], a measure of wellbeing for older adults. Both measures can be used to assess care-dependent people’s QoL in cross-sectional studies, but require more complex designs (e.g. longitudinal data collection in randomized controlled trials (RCTs)) to evaluate the *impact* of LTC service provision on people’s lives.

The Adult Social Care Outcomes Toolkit (ASCOT) was developed in England to measure the *QoL-effects* of LTC service provision [4, 5]. ASCOT aims to be useful for settings not suitable for RCTs as it evaluates the impact of LTC service provision on QoL by comparing LTC-related QoL (also referred to as social care-related QoL (SCRQoL) in the English version) in the presence of care services with LTC-QoL in the hypothetical absence of services. Since its development in 2010, ASCOT has gained interest in a number of English and non-English speaking countries. It has been translated for studies in Denmark, Italy, Finland, the Netherlands [6] and, recently, Japan [7]. So far, the ASCOT instrument for service users had not been translated into German.

German speaking countries comprise a large and several smaller countries with a substantial number of long-term care recipients in total. In 2017, about 3.4 million people relied on long-term care in Germany; of which 1.6 million received publicly co-funded long-term care services either at home or in care homes [8]. In Austria, a country with about a tenth of Germany’s population, some 460,000 people were eligible for long-term care allowance, a universal benefit for people in need of care in 2017, with 240,000 home care services users, visitors of care centers or residents in care homes [9]. Seventy percent of the population in Switzerland, a country of about the same population size as Austria, speak German. In 2017, some 500,000 people in Switzerland received home care or lived in a care home [10]. In addition, in Europe, German is the co-official language in Liechtenstein, Luxemburg (besides French and Luxemburgish), Belgium (besides Dutch and French) and in dependent entities of Italy and Poland.

The aim of this study was to translate and culturally adapt the ASCOT interview version (INT4) for LTC service users into German and to test the content and construct validity of the German translation for both the current and expected QoL states. Cross-cultural adaption captures the translation itself and the cultural adaptation to develop an instrument to be used in another setting [11]. Cross-cultural validity refers to whether the meanings (semantic), content interpretations and concepts of the instrument in different languages/cultures are similar enough [12] such that there are no differences in substantive meaning and measurement between the original and the translated tool [13]. In this paper, the investigation of the validity of the German version of ASCOT considers the linguistic, content, and construct validity of the translated instrument in relation to the original toolkit in English. The assessment results also give insight into challenges for translation, solutions to these challenges, and provides evidence on the extent of cross-cultural equivalence. A cross-culturally valid German adaptation of the ASCOT service user measure can be included in data collection on outcomes of LTC service provision to inform national policy makers, care organization managers, and researchers in German-speaking countries and to enable cross-national comparative studies on LTC-outcomes.

## Methods

### The ASCOT service user instrument

The ASCOT instruments for measuring self-assessed quality-of-life effects of care service provision are available as an interview version (INT4) and a self-completion tool (SCT4). Both tools address eight distinct QoL-domains of LTC service users (Table 1), covering basic (domains 2–4 and 7) and higher order aspects (1 and 5–6) of LTC-QoL. Additionally, the *Dignity* domain (8) captures how LTC services affect service users’ self-esteem [4].

**Table 1:** The domains of the ASCOT service user instrument

Domain	Definition
1. Control over daily life	The service user can choose what to do and when to do it, having control over his/her daily life and activities
2. Personal cleanliness and comfort	The service user feels he/she is personally clean and comfortable and looks presentable or, at best, is dressed and groomed in a way that reflects his/her personal preferences
3. Food and drink	The service user feels he/she has a nutritious, varied and culturally appropriate diet with enough food and drink he/she enjoys at regular and timely intervals
4. Personal safety	The service user feels safe and secure. This means being free from fear of abuse, falling or other physical harm
5. Social participation and involvement	The service user is content with their social situation, where social situation is taken to mean the sustenance of meaningful relationships with friends and family and feeling involved or part of a community - should this be important to the service user
6. Occupation	The service user is sufficiently occupied in a range of meaningful activities whether it be formal employment, unpaid work, caring for others or leisure activities
7. Accommodation cleanliness and comfort	The service user feels their home environment, including all the rooms, is clean and comfortable
8. Dignity	The negative and positive psychological impact of support and care on the service user’s personal sense of significance

Source: Netten et al. [4]

Each domain has four response options covering states that reflect people’s wishes (ideal state, coded 3), ‘must not grumble’ situations (no needs, coded 2), reduced quality-of-life states (some needs, coded 1) and situations in which physical or mental health is affected or soon will be (high needs, coded 0) [4]. The LTC-QoL is the total raw score of the eight domains – ranging from 0 (worst state) to 24 (ideal state).

The ASCOT INT4 assesses the QoL-impact of LTC services by capturing two self-estimated states for each domain: the ‘current LTC-QoL state’, with the LTC services in place, and the ‘expected LTC-QoL state’ in the hypothetical absence of LTC services [4], allowing an estimation of the counter-factual for settings where RCTs are not possible or difficult to implement [5, 14, 15]. The difference between the two LTC-QoL states reflects care service-induced changes in QoL of service users. In addition to the raw score, a preference-weighted ASCOT score reflects the *relative importance* of outcomes in the individual QoL-domains in a country. While preference-weights for other German-speaking countries remain to be elicited, preference-weighted ASCOT scores for Austria can be generated by using the population-based weights [16].

From a conceptual perspective, ASCOT can be assigned to the outcome measures following a so-called ‘formative measurement model’ [17], as a latent construct (here service users’ LTC-QoL or SCRQoL in the original English version) is formed by a combination of its items (here eight ASCOT domains). Variation in service users’ LTC-QoL result from variation in the ASCOT domains. Thus, these eight domains define the latent construct and are not interchangeable. Adding or dropping an ASCOT domain may affect the conceptual interpretation of the LTC-QoL-construct. Contrary to items in ‘reflective measurement models’, items in formative models do not share a singular common theme. The measurement model type has implications for the presentation of scores, as in formative models a weighted combination of the items better reflects each item’s unique contribution to the latent construct [18]. In addition, the type of measurement model has implications for the assessment of measurement properties [18, 19].

### Translation and cultural adaptation into German

The ASCOT service user instruments (SCT4 and INT4) were translated from English into German between June 2015 and March 2016 by the Austrian research team in cooperation with the translation agency PharmaQuest (now part of Corporate Translations, Inc.) and the ASCOT development team in England. The Austrian team also involved colleagues from Germany to establish an appropriate translation for both German-speaking countries.

**Fig. 1:**
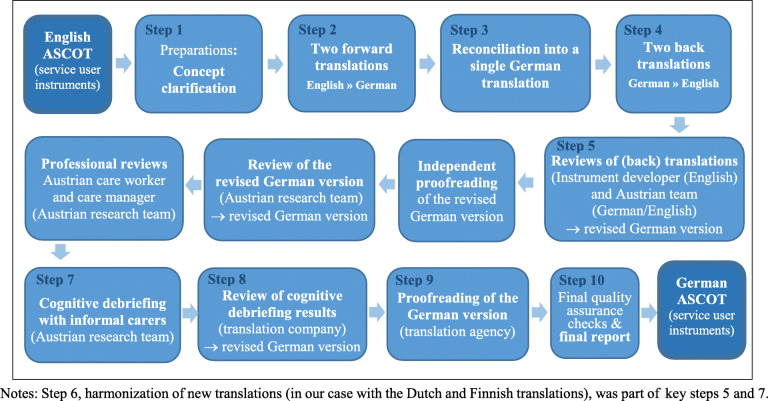
Flow diagram of the translation and cross-cultural adaptation process of ASCOT service user instruments, SCT4 and INT4 (English into German). Source: PharmaQuest Ltd., authors’ illustration

Figure 1 describes the German translation and cultural adaptation of the English ASCOT service user instruments (INT4 and SCT4). It followed step by step the ISPOR’s principles of good practice for the translation and cultural adaptation (TCA) process for patient-reported outcome (PRO) measures [20]. Based on the ASCOT concept clarification guide (Step 1) provided by the ASCOT development team, two forward translations (English into German) were conducted by two independent translators (Step 2). The English-to-German translations were consolidated into one (Step 3), then translated back into English (Step 4) and reviewed by the translation agency, the ASCOT development team and the German-speaking team of researchers (Step 5). Step 6 of the ISPOR TCA guideline suggests comparing the translation with previous translations into other languages. We reviewed the Dutch translation of the ASCOT service user instrument [21] and shared our experiences in Step 4 and 7 of the ISPOR TCA process with our Finnish colleagues who worked at a Finnish translation as part of the same project [22]. In addition to the ISPOR TCA key steps, the German version was proofread by an independent translator and reviewed by the German-speaking research team, a professional care worker and a care manager before pilot testing. After cognitive debriefings with six LTC service users (Step 7), comments were sent to the translation agency for review (Step 8). The pre-final version was proofread (Step 9) before the translation agency approved the German ASCOT instrument for service users (Step 10). The complete original English version and the final German versions of the ASCOT service user instruments are available on the ASCOT website (http://www.pssru.ac.uk/ascot or https://short.wu.ac.at/ascot).

### Data collection and methods for the analysis

#### Data collection

We used two data sources to assess forms of validity of the German version of the ASCOT for service users. First, cognitive debriefing interviews, conducted as part of the German translation and adaptation process, provided insights into the understanding of questions and response options of the ASCOT INT4 and SCT4 versions. Six cognitive interviews with LTC service users (four women, two men) were carried out in November and December 2015. The respondents were recruited from two major LTC service providers in Vienna. The three interviewers were members of the Austrian research team (two women, one man) who were trained in conducting cognitive debriefings with the target group, familiar with the ASCOT instrument and provided with a comprehensive interview guide. After each interview, experiences were exchanged in the team. The cognitive debriefing interviews with LTC service users were conducted using the think-aloud method and verbal probing techniques [23, 24].

Second, the final version of the German ASCOT instrument (INT4) for service users was used in a survey in Austria. In total, 633 LTC service users were interviewed across all nine Austrian regions ('Laender') via computer-aided personal standardized interviews (CAPI) using the online survey software ‘Qualtrics’.^1^ The electronic format allowed us to apply measures to improve data quality. Piping previous responses into questions or response options helped to personalize the interview, avoid inconsistencies and to reduce the survey burden for both interviewees and interviewers. Questions were grouped per topic to enable a good flow of questions using filter questions. In order to decrease the risk of data loss due to no or poor connectivity, an app with an offline functionality was used.

**Fig. 2:**
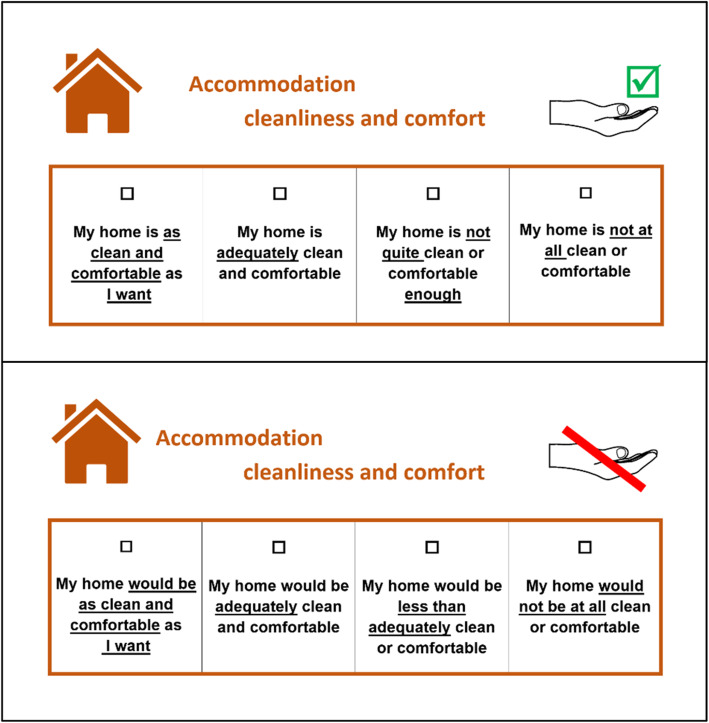
Sample showcards for response options of the ASCOT domain “Accommodation cleanliness and comfort”, current QoL state (with care services) and expected QoL state in absence of services (English version). Note: the showcards were designed for the collection in German (https://short.wu.ac.at/ascot); the example above uses the wording of the response options of the English version of ASCOT-INT4 [4]

Measures to facilitate interviewing and to reduce potential interviewer bias addressed the recruitment (e.g. interviewers recruited spoke the regional dialect), the training and the supervision. The interviewer training sessions covered the aims and methods of data collection, a briefing on interviewing older people and on the details on the LTC services for the specific region. Showcards were prepared for the German ASCOT questionnaire, displaying the response options for the current and expected QoL-states in large print (18 pt.) on one landscape page each (see Fig. 2 in the Appendix for the two showcards for a sample domain). All interviewers were asked to contact the Austrian research team to report on their experiences after each interview. For information on the data collection see [25].

#### Assessing the validity of the German version of ASCOT for service users

Table 2 gives an overview of the key concepts investigated in the analysis, data and methods used for assessing the validity of the German version of ASCOT for service users. Linguistic and content validity was evaluated using cognitive interview transcripts. For both types of validity, responses and statements of the interviewees were assessed for equivalence with the concepts and constructs used in the ASCOT concept clarification guide. Construct validity was tested using survey data.

**Table 2:** Overview of the key concepts, data and methods for assessing cross-cultural adaptation of the German ASCOT instrument for service users

Concept	Aim	Data/Tools	Method
Linguistic validity	Establish conceptual and linguistic equivalence between the original and translated version of the instrument, its survey questions, and response choices [13].	Cognitive interview transcripts	Analysis of the interview transcripts to identify discrepancies in the meaning of the translated ASCOT items and response options from the original concepts. Analysis was based on the individual item and consisted of summarizing responses, identifying problem areas and suggesting improvements where needed [26].
Content validity	Assure that the content of an instrument is an adequate reflection of the constructs intended to be measured [27] and whether the instrument, its components (items), and response choices are comprehensive, understandable and acceptable [28].	Cognitive debriefing interview transcripts; ASCOT concept clarification guide [4, 20]	Analysis of the interview transcripts to assess whether the translation reflected the same item content as the original version, the explanations of responses were compared to the ASCOT concept clarification guide.
Construct validity	Investigate how similar the instrument is to other conceptually-related measures (convergent validity) or whether the instrument is able to differentiate where differences between groups are anticipated (know-group validity or discriminative validity) [27] [29].	Survey data (*n* = 633)	Hypothesis testing using bivariate tests of association with related constructs and service user characteristics.

Following the COSMIN (COnsensus-based Standards for the selection of health Measurement INstruments) checklist [29, 30], construct validity of the translated instrument was assessed using hypothesis testing. We applied a series of bivariate association tests to evaluate whether the translated ASCOT instrument measures the intended constructs (both overall and per domain). We explored convergent validity using related constructs and known-group validity using service user characteristics reflecting the intensity of need and care service process characteristics. In addition, we investigated whether the German translation performed similarly to the English original by looking at previous empirical evidence on construct validity published for the original instrument [31, 32] and the validation of translations [21] and checking whether the relationships established there could also be found for the German ASCOT. Table 3 summarizes the expected associations.

**Table 3:** Anticipated associations with the German ASCOT scores and domains

Variables	Hypotheses	Anticipated associations (for the current and expected ASCOT states)
**Well-Being & Health**		
Self-perceived QoL		*Current state*:
	H1	Positive correlation between the *current overall ASCOT score* and self-rated QoL [32]
	h1	Positive associations of QoL with *all ASCOT domains* [31]
		*Expected state:*
	H6	Positive, but weak correlation between QoL and *expected overall ASCOT score* in the absence of LTC services as the expected state does not capture the compensatory effect of services (whereas the QoL item relates to the current situation, i.e. with services)
EQ-5D		*Current state:*
	H2	Weak to moderate positive correlation of the EQ-5D index with *current overall ASCOT score* [32]
	h2	Positive association of the EQ-5D index with *all ASCOT domains*
	h-cont1	Positive associations between EQ-5D items (self-care and usual activities) and *Control over daily life* as being able to perform those tasks could lead to higher perceived control over daily life [31]
	h-clean1	Positive association between EQ-5D item self-care and ASCOT *Personal cleanliness,* as self-care (washing and dressing) is related to feelings of personal cleanliness [31]
	h-safe1	Positive associations between all EQ-5D items (mobility, self-care, usual activities, pain, and anxiety/depression) and *Personal safety* as they capture factors inside and outside the home that could make a person feel unsafe [31]
	h-occu1	Positive association between EQ-5D item usual activities and ASCOT *Occupation*, as performing usual activities is related to being able to do things one enjoys [31]
		*Expected state*:
	H5	Positive correlation is expected between the EQ-5D index and the *overall expected ASCOT score* in the absence of LTC services, as it measures limitations regardless of service effects
**Functioning**		
(Instrumental) activities of daily living, (I)ADLs		*Current state*:
	H3	Negative correlation between the (I)ADL limitations index and the *current overall ASCOT score,* as higher needs will affect QoL if services cannot fully compensate [31, 32]
		*Expected state*:
	H4	Stronger negative correlation between (I)ADL limitations index and the *expected ASCOT score* (compared to the current score) as (I)ADLs are a measure of needs [5]
	h4	Negative associations between the (I)ADL limitations index and *all expected ASCOT domains*
	h-cont2	As limitations in the ability to cope with (I)ADLs may come with feelings of less control, we expect negative associations between all (I)ADLs and *Control over daily life* (*expected)*
	h-clean2	Negative associations between ADLs linked to personal care (washing, bathing, toilet, dressing) and *Personal cleanliness (expected)*
	h-food1	Negative associations between food-related (I)ADLs (eating, shopping) as well as ‘using the toilet’ [31]) and *Food and drink (expected)*
	h-acco1	Negative associations between housework IADL and *Accommodation (expected)*
	h-safe2	Negative associations between (I)ADLs that require a certain amount of physical mobility/walking around (getting in and out of bed, showering/bathing, getting around, stairs, getting out of the house, shopping, housework) and *Personal safety (expected)*
	h-safe3	Negative association between (I)ADLs requiring mobility or being outdoors, as well as taking medicine and *Personal safety (expected)*
	h-occu2	Negative association between all (I)ADLs and *Occupation (expected)*
**Control and autonomy**		
Control and Autonomy subscale of CASP-12		*Current state:*
	H8	Higher *current overall ASCOT score* for persons scoring high on the Control and Autonomy subscale of CASP-12 [32]
	h3	Positive associations between CASP-12 and *all ASCOT domains* [31]
**Environment**		
Design of home		*Current state:*
	h-cont3	Positive association with *Control over daily life* [31]
	h-clean3	Positive association with *Personal cleanliness* [31]
	h-acco2	Positive association of design of home with *Accommodation* [31]
	h-safe4	Positive association with *Personal safety* as good home design makes it easier to provide appropriate care at home, resulting in higher QoL for these domains [31]
Interviewer-assessed cleanliness of home	h-acco3	Positive association with *Accommodation* [31]
Getting around locally	h-cont4	Positive association with *Control over daily life* [31]
	h-safe5	Positive association with *Personal safety*
	h-soci1	Positive associations with *Social participation* [31], since local accessibility enables users to achieve (good) outcomes in this domain
	h-occu3	Positive association with *Occupation* [31]
**Social Contact/Support**		
Speak to relatives/friends on phone, speak to neighbors, meet up with relatives/friends		*Current state*:
	h-soci2	Positive association between contact with people outside of the home and *Social participation* [31]
**Care Service Process Quality**		
Process quality index score		*Current state*:
	H7	Higher *current overall ASCOT score* for persons with higher process quality ratings [32]
	h-dig1	Positive association with the ASCOT *Dignity* domain [31, 32]
Overall satisfaction with services	h-dig2	Positive association with the ASCOT *Dignity* domain [31, 32]

Notes: H indicates hypotheses related to the ASCOT score; h indicates hypotheses related to *all* ASCOT items; h-clean/occu/social etc. indicates hypotheses related to a specific ASCOT domain, such as personal cleanliness, occupation, social participation etc. The enumeration (1, 2, 3, …n) uniquely identifies either the hypothesis related to the ASCOT score, e.g. H1, or the hypothesis related to a specific domain, e.g. h-clean1

The variables used for testing the hypothesized relationships cover different areas. In terms of well-being and health measures, we used self-perceived quality of life (QoL) as a seven-point measure (categories were collapsed for certain analysis), and health-related quality of life, measured by individual EQ-5D-3L items as well as the EQ5D index, anchored at 0 for death and 1 for perfect health, using German weights [2, 33]. For assessing individuals’ functioning, we used individual (I)ADLs ((instrumental) activities of daily living [34, 35]) and an (I)ADL limitations index [34, 35], with 0 reflecting no (I)ADL issues (person is able to perform all (I)ADLs by themselves) and 39 indicating the highest level of impairment (person is not able to do any of the (I)ADLs). To measure control and autonomy, the relevant items from the CASP-12 scale were combined to form an index [36] ranging from 0 (no control/autonomy) to 18 (highest level of control/autonomy). We also looked at selected aspects of the environment, such as home design (interviewer-rated, self-rated), home cleanliness, and accessibility of the local area. Social contact was assessed via a series of questions on the frequency of social contact (how often respondents speak to relatives or friends on the phone, speak to neighbors or meet up with relatives or friends in person). Lastly, care service process quality was measured using a seven-point single-item rating of satisfaction with services and a process quality index consisting of several service process characteristics [37] (such as whether care workers arrive on time or spend enough time with the service user), ranging from 0 to 34, with 0 indicating the worst possible rating of care service processes.

We used the Spearman correlation coefficient to assess associations between the ASCOT score and related constructs and Fisher’s exact tests (for categorical variables) and one-way analyses of variance (for continuous variables) to test the hypothesized associations for each ASCOT domain. Known-groups or discriminative validity was explored by performing t-tests comparing the ASCOT scores between previously specified subgroups of the sample. Domain-wise Benjamini and Hochberg [38] correction for multiple testing was applied where needed (i.e., where several consecutive tests were carried out per domain). All statistical analyses were conducted in Stata v15 [39].

We applied a two-step process: first, we tested the correlations between the current and expected ASCOT *scores* and related constructs to check for the general relationships (Table 3). Building on Rand et al. [32], Malley et al. [40] and Malley et al. [5], we expected the *current* ASCOT score to be positively correlated with other QoL scores, such as general QoL measure (H1) and health-related quality of life measured by EQ-5D (H2), and negatively correlated with limitations in (instrumental) activities of daily living ((I)ADLs) (H3). With regard to the second ASCOT score, aiming to capture *expected* QoL states in the absence of LTC services, we assumed a negative correlation with indicators of impairments, such as the (I)ADL limitations index (H4), a positive correlation with the EQ5D-index (H5) and a weak positive correlation with general QoL (H6) [4, 5, 31, 32]. Furthermore, we expected the current ASCOT score to differ between groups of service users who differed in terms of care service process quality assessment (H7), and perceived control and autonomy (CASP-12 subscore) (H8).

Second, we used hypothesis testing to assess construct validity for *each of the* ASCOT *domains* (Table 3). We mainly focused on the current states for validation, as the expected state represents a hypothetical situation that can only be approximated via other measures, but not directly assessed. For the expected QoL states of the domains, we checked for associations with selected (I)ADL restrictions, as these can give insight into how an individual would perform in the absence of services [5]. The levels of significance are in accordance with the threshold values of the validation of the original English ASCOT [31].

## Results

### Sample characteristics

#### Sample for the cognitive interviews

Six LTC service users (four women, two men) took part in the cognitive debriefing. The youngest participant was 55 years, the oldest 85 years. Experiences with care services ranged from 6 months to 15 years, with 5 respondents using care services up to 5 years.

#### Survey sample

Table 4 shows the survey sample characteristics. The Austrian sample of home care service users was representative of national data [41, 42] with respect to gender, age and care needs, indicated by the LTC allowance level (1–7), a cash benefit granted to people in need of LTC, irrespective of income. 67% of the 633 LTC service users in the Austrian sample were women. Respondents below the age of 60 comprised 4% of the sample. Almost a quarter were aged 60–74 and more than a third of the sample each were aged 75–84 or 85 or older. 46% of those who participated in the study had substantial care needs as indicated by LTC allowance level 3 to 7, which means they needed support for more than 120 h per week.

**Table 4:** Sample characteristics: LTC service user survey participants

	n	%
**Age group**		
55–64	59	9.32
65–74	116	18.33
75–84	230	36.33
85 and older	228	36.02
**Sex**		
Female	425	67.14
Male	208	32.86
**Years using care services at home**		
Less than 1 year	107	17.04
1 year to less than 2 years	120	19.11
2 years to less than 5 years	249	39.65
5 years to less than 10 years	104	16.56
10 years or more	48	7.64
**LTC allowance level**		
No LTC allowance	43	6.79
Level 1	127	20.06
Level 2	156	24.64
Level 3	119	18.80
Level 4	111	17.54
Level 5	62	9.79
Level 6	4	0.63
Level 7	3	0.47
Missing (care level not known or not yet assessed)	8	1.26
**TOTAL**	**633**	**100.00**

Source: WU, EXCELC INT SU AUT 2016/2017

The distribution of responses for each ASCOT domain is shown in Table 5 and the distributional statistics for current and expected overall ASCOT score are shown in Table 6.

**Table 5:** Responses to the German ASCOT questionnaire for home care service users (*n* = 633)

		CONTROL OVER DAILY LIFE	PERSONAL CLEANLINESS	FOOD AND DRINK	ACCOMMODATION	PERSONAL SAFETY	SOCIAL PARTICIPATION	OCCUPATION	DIGNITY
	Frequencies (%)								
Current ASCOT Score	High needs	34 (5.4)	1 (0.2)	7 (1.1)	3 (0.5)	21 (3.3)	48 (7.6)	13 (2.1)	4 (0.6)
	Some need	141 (22.3)	18 (2.8)	24 (3.8)	42 (6.6)	95 (15.0)	124 (19.6)	107 (16.9)	53 (8.4)
	No needs	191 (30.2)	203 (32.1)	180 (28.4)	216 (34.1)	255 (40.3)	216 (34.1)	197 (31.1)	143 (22.6)
	Ideal state	267 (42.2)	411 (64.9)	418 (66.0)	370 (58.5)	260 (41.1)	242 (38.2)	311 (49.1)	424 (67.0)
	Missing	0 (0.0)	0 (0.0)	4 (0.6)	2 (0.3)	2 (0.3)	3 (0.5)	5 (0.8)	9 (1.4)
Expected ASCOT Score	High needs	254 (40.1)	147 (23.2)	156 (24.6)	129 (20.4)	131 (20.7)	121 (19.1)	96 (15.2)	n.a
	Some need	217 (34.3)	178 (28.1)	117 (18.5)	220 (34.8)	173 (27.3)	175 (27.7)	175 (27.7)	n.a
	No needs	83 (13.1)	136 (21.5)	110 (17.4)	148 (23.4)	172 (27.2)	155 (24.5)	156 (24.6)	n.a
	Ideal state	67 (10.6)	151 (23.9)	226 (35.7)	125 (19.8)	142 (22.4)	169 (26.7)	185 (29.2)	n.a
	Missing	12 (1.9)	21 (3.3)	24 (3.8)	11 (1.7)	15 (2.4)	13 (2.1)	21 (3.3)	n.a

Source: WU, EXCELC INT SU AUT 2016/2017

**Table 6:** Distributional statistics for current and expected ASCOT score of the German version

	German ASCOT Scores (range: 0–24)				
	mean (SD)	median	min	max	n
Current ASCOT Score	18.92 (3.38)	19	6	24	612
Expected ASCOT Score	12.97 (5.21)	13	1	24	569

Source: WU, EXCELC INT SU AUT 2016/2017

### Linguistic and content validity of the German version of ASCOT

The cognitive interviews conducted as part of the translation process showed that the German ASCOT domains were generally understood as intended. We found good evidence to support content validity for six out of eight ASCOT domains: *Personal cleanliness*, *Food and drink*, *Personal safety*, *Social participation*, *Occupation* and *Accommodation*. Particularly, the *Personal cleanliness* and *Accommodation* domains were understood very well.

Questions and response options for two domains, *Control over daily life* and *Dignity,* appeared difficult to translate into German. Literal translations did not work as they did not convey the same meaning and are not commonly used in everyday German language. For *Control over daily life*, the literal translation would have sounded too strict. Therefore, a different wording was chosen which translated back into ‘being able to influence daily life’, for which two wording choices were tested to stress the extent of independency: ‘nach eigenem Ermessen’ (at one’s own discretion) and ‘selbstbestimmt’ (self-determined). Both translations were understood by LTC service users; however, the interviewees clearly preferred the latter translation as it more clearly reflected its use in everyday language. Similarly, the question and response options for the *Dignity* domain (to ‘think and feel about oneself’) could not be translated literally as the German speaking LTC service users did not like to talk in this way about themselves. Since the translation into Dutch, a language related to German, experienced similar challenges for the *Dignity* domain [21], we changed the wording to ‘how having help affects your self-esteem’.

In addition to the amendments in the two domains *Control over daily life* and *Dignity*, we made some adaptations during the translation process in discussion with the ASCOT development team to improve the conceptual equivalence between the original and translated version of ASCOT. One of these changes referred to the translation of the domain *Personal cleanliness and comfort* that captures two concepts, ‘personal cleanliness’ and ‘comfort’*.* We added ‘körperlich’ (‘physical’) to ‘Wohlbefinden’ (‘comfort’) to make sure the concept will be understood as intended.

Careful wording was needed for English adjectives, such as ‘adequate’, which are also used in German but literal translation did not reflect the same meaning. We specified the meaning of ‘adequate’ to ensure that everyone understands it in the same way and changed the translation of ‘adequate’ food and drink to ‘enough and appropriate’ food and drink. This corresponds with the Dutch translation as ‘adequate’ was translated as ‘sufficient/enough’ [21].

### Construct validity of the German version of ASCOT

#### Construct validity of the overall ASCOT score (German version)

Table 7 shows significant associations between the overall ASCOT scores (current and expected) and related outcome scales. All correlation coefficients are lower than 0.5, suggesting a moderate correlation [43]. As expected, both QoL-related measures, i.e. self-rated quality of life (H1) and the EQ-5D index (H2), were significantly and moderately related with the ASCOT scores. Self-rated QoL was positively correlated with the expected ASCOT score (H6) but to a lower extent than with the current ASCOT score. On the other hand, EQ-5D index showed a stronger positive correlation with the expected ASCOT score (H5) than with the current score (H2). Limitations in (I)ADLs are frequently used as a measure of ‘need’ and disability [31]. As expected, the (I)ADL-score was negatively (H3) but weakly (< 0.3) correlated with the *current* overall ASCOT score, indicating that (I)ADLs do not capture the compensatory activity of LTC services, reflected in the current ASCOT score. The (I)ADL limitations index was stronger correlated with the *expected* ASCOT score (H4) than with the *current* ASCOT score. Thus, the (I)ADL limitations index better captured abilities of service users and the QoL of service users if they had no care services (Table 7).

**Table 7:** Relationship between the German ASCOT score and related outcome measures and service user subgroups

		Current ASCOT score		Expected ASCOT score	
		Spearman’s rho (Sign.)	n	Spearman’s rho (Sign.)	n
QoL		0.363 ***	608	0.225 ***	566
EQ-5D index		0.345 ***	598	0.425 ***	556
(I)ADL limitations index		−0.225 ***	573	−0.425 ***	538
		**Mean**	**n**		
CASP-12 subscore (autonomy & control)^a^	Low	17.87	306		
	High	20.22	273		
	t-value (sign.)	−8.89 ***			
Service quality index^a^	Low	18.26	282		
	High	19.73	213		
	t-value (sign.)	−4.84 ***			

Source WU, EXCELC INT SU AUT 2016/2017

Notes: ***significant at 1% level; ^a^cut-off determined by median split

**Table 8:** Relationship between the German version of the ASCOT domains (current states) and related constructs

			Self-rated QoL^**1**^	EQ-5D index^**2**^	CASP-12’s autonomy and control subscore^**2**^
ASCOT domains	Response levels	n	% (very) good	mean (sd)	mean (sd)
**CONTROL OVER DAILY LIFE**	High needs	34	38.24	0.38 (0.28)	10.63 (3.67)
	Some needs	141	43.57	0.41 (0.27)	10.86 (3.09)
	No needs	191	60.21	0.55 (0.28)	11.77 (3.27)
	Ideal state	267	70.08	0.61 (0.27)	12.69 (3.22)
	F stat (Sign.)		***	20.22 ***	11.37 ***
**PERSONAL CLEANLINESS** ^a^	High needs	1	44.44	0.42 (0.33)	11.18 (4.10)
	Some needs	18			
	No needs	203	51.23	0.48 (0.28)	11.06 (3.04)
	Ideal state	411	64.22	0.57 (0.28)	12.35 (3.33)
	F stat (Sign.)		***	7.9 ***	10.37 ***
**FOOD AND DRINK** ^a^	High needs	7	29.03	0.36 (0.30)	9.07 (3.19)
	Some needs	24			
	No needs	180	56.18	0.50 (0.28)	11.24 (2.91)
	Ideal state	418	63.22	0.57 (0.28)	12.42 (3.34)
	F stat (Sign.)		***	9.98 ***	20.32 ***
**ACCOMMODATION** ^a^	High needs	3	31.11	0.39 (0.28)	9.86 (3.41)
	Some needs	42			
	No needs	216	54.67	0.54 (0.28)	11.14 (3.06)
	Ideal state	370	65.76	0.55 (0.29)	12.56 (3.26)
	F stat (Sign.)		***	6.84 ***	22.52 ***
**PERSONAL SAFETY** ^a^	High needs	21	40.00	0.26 (0.21)	11.05 (4.01)
	Some needs	95		0.40 (0.27)	10.38 (2.83)
	No needs	255	60.63	0.58 (0.26)	11.59 (3.21)
	Ideal state	260	67.05	0.58 (0.29)	12.84 (3.26)
	F stat (Sign.)		***	18.79 ***	14.91 ***
**SOCIAL PARTICIPATION**	High needs	48	37.50	0.43 (0.29)	10.68 (3.20)
	Some needs	124	47.54	0.47 (0.29)	10.75 (3.15)
	No needs	216	57.87	0.55 (0.27)	11.52 (3.14)
	Ideal state	242	71.67	0.58 (0.28)	13.03 (3.23)
	F stat (Sign.)		***	6.99 ***	17.97 ***
**OCCUPATION** ^a^	High needs	13	37.82	0.27 (0.24)	10.45 (2.77)
	Some needs	107		0.45 (0.28)	10.28 (3.16)
	No needs	197	58.88	0.54 (0.27)	11.38 (3.16)
	Ideal state	311	68.51	0.58 (0.28)	12.84 (3.20)
	F stat (Sign.)		***	9.74 ***	19.66 ***
**DIGNITY** ^a^	High needs	4	35.09	0.43 (0.26)	9.65 (2.73)
	Some needs	53			
	No needs	143	59.86	0.56 (0.30)	12.04 (3.33)
	Ideal state	424	62.23	0.55 (0.28)	12.13 (3.27)
	F stat (Sign.)		***	4.47 **	14.19 ***

Notes: ^a^ lowest two levels of the ASCOT domain are collapsed because of small numbers; ^1^ Fisher’s exact test, ^2^ one-way analysis of variance

***significant at 1% level, ** significant at 5% level

Source: WU, EXCELC INT SU AUT 2016/2017

In order to assess known-groups or discriminative validity, we compared ASCOT scores across two previously specified groups of LTC service users (Table 7). We checked for differences between persons with low and high self-perceived control and autonomy according to the relevant CASP-12 items, and low and high service satisfaction (both overall and measured by a service quality index). *Current* ASCOT scores were significantly higher in persons with higher perceived control and autonomy as measured by the CASP-12 subscale (H8). Persons with a more positive service experience (higher values in the service process quality index (H7)) also had significantly higher *current* ASCOT scores.

#### Domain-specific construct validity of the ASCOT service user instrument (German version)

Tables 8, 9 and 10 show the results for the evaluation of the construct validity of all ASCOT domains: as hypothesized, overall QoL (h1), the EQ-5D index (h2) and the control-and-autonomy subscale of the CASP-12 (h3) were significantly and positively associated with *all* of the current ASCOT domains (Table 8), which is in line with results from the English ASCOT instrument [31].

**Table 9:** Relationship between the German version of the ASCOT domains (Food and Drink excluded) for current LTC-QoL states with care services and related constructs and service user groups

**ASCOT Domains**		**CONTROL OVER DAILY LIFE with LTC services**					**PERSONAL CLEANLINESS with LTC services**					**ACCOMMODATION with LTC services**				
	**Response Levels**	**High Needs**	**Some Needs**	**No Needs**	**Ideal State**	**Sign.**	**High Needs**	**Some Needs**	**No Needs**	**Ideal State**	**Sign.**	**High Needs**	**Some Needs**	**No Needs**	**Ideal State**	**Sign.**
	**n**	**34**	**141**	**191**	**267**		**1**	**18**	**203**	**411**		**3**	**42**	**216**	**370**	
**Well-Being & Health**																
EQ-5D 2: self-care	% moderate / extreme problems	94.12	81.56	75.13	56.77	***	77.38 ^a^			65.77	***					
EQ-5D 3: usual activities		94.12	90.71	82.01	70.83	***										
**Environment**																
Design of home	% meets most/ all needs	79.41	87.23	90.05	96.21	***	86.88 ^a^			93.89	***	73.33		87.96	95.64	***
Interviewer assessed cleanliness of home	% (very) clean											51.11		75.6	91.01	***
Getting around locally	% to all places	24.14	17.78	26.26	42.86	***										
**ASCOT Domains**		**PERSONAL SAFETY with LTC services**					**SOCIAL PARTICIPATION with LTC services**					**OCCUPATION with LTC services**				
	**Response Levels**	**High Needs**	**Some Needs**	**No Needs**	**Ideal State**	**Sign.**	**High Needs**	**Some Needs**	**No Needs**	**Ideal State**	**Sign.**	**High Needs**	**Some Needs**	**No Needs**	**Ideal State**	**Sign.**
	**n**	**21**	**95**	**255**	**260**		**48**	**124**	**216**	**242**		**13**	**107**	**197**	**311**	
**Well-Being & Health**																
EQ1: mobility	% moderate/ extreme problems	95.65 ^a^		90.08	83.92	***										
EQ2: self-care		81.03 ^a^		72.83	62.02	***										
EQ3: usual activities		91.30 ^a^		80.31	74.32	***						95.80 ^a^		83.16	71.52	***
EQ4: pain		97.41 ^a^		84.98	81.08	***										
EQ5: anxiety		74.14 ^a^		55.73	40.23	***										
**Social Contact/Support**																
Speak to relatives/ friends on phone	% weekly						62.50	76.42	81.48	85.83	***					
Speak to neighbors							39.58	44.26	58.80	64.17	***					
Meet up with relatives/friends							18.75	46.34	58.33	64.32	***					
**Environment**																
Design of home	% meets most/all needs	82.76 ^a^		91.73	94.98	***										
Getting around locally	% to all places	13.21 ^a^		32.64	37.40	***	20.93	18.75	25	44.93	***	17.92 ^a^		28.19	38.38	***

Notes: Fisher’s exact test, ^a^ lowest two levels of the ASCOT domain are collapsed, except for Personal cleanliness where lowest three levels are collapsed

*** significant at 1% level, Benjamini & Hochberg correction for multiple testing applied

Source: WU, EXCELC INT SU AUT 2016/2017

**Table 10:** Relationship between the German version of the ASCOT domains for the expected LTC-QoL states in absence of services and functional abilities: (instrumental) activities of daily living ((I)ADLs)

**ASCOT Domains**		**CONTROL OVER DAILY LIFE without LTC services**					**PERSONAL CLEANLINESS without LTC services**					**FOOD AND DRINK without LTC services**					**ACCOMMODATION without LTC services**				
**Response Levels**		**High Needs**	**Some Needs**	**No Needs**	**Ideal State**	**F stat Sign.**	**High Needs**	**Some Needs**	**No Needs**	**Ideal State**	**F stat Sign.**	**High Needs**	**Some Needs**	**No Needs**	**Ideal State**	**F stat Sign.**	**High Needs**	**Some Needs**	**No Needs**	**Ideal State**	**F stat Sign.**
	**n**	**254**	**217**	**83**	**67**		**147**	**178**	**136**	**151**		**155**	**117**	**110**	**226**		**114**	**210**	**141**	**116**	
(I)ADL limitations index, mean (sd)		17.33 (8.04)	12.57 (6.85)	11.2 (7.71)	9.09 (6.96)	30.65***	19.8 (7.95)	15.25 (6.93)	11.96 (6.5)	8.55 (6.11)	67.96***	18.03 (7.94)	13.89 (6.63)	13.12 (6.98)	11.67 (8.24)	20.35***	16.89 (8.19)	11.6 (7.04)	13.38 (7.61)	15.57 (8.74)	13.71***
Getting in and out of bed	% can only do with help	31.5	11.98	14.46	4.48	***															
Washing hands and face		17.32	3.69	6.02	2.99	***	22.45	9.55	2.09^a^		***										
Taking a bath or shower		79.13	62.5	57.83	35.82	***	89.73	84.27	54.41	32.45	***										
Dressing or undressing		53.54	30.41	27.71	22.39	***	65.31	43.82	29.41	14.57	***										
Using the toilet		28.85	8.76	13.25	5.97	***	41.1	12.92	10.29	5.30	***	30.32	11.11	11.82	14.6	***					
Eating, including cutting up food		22.05	12.44	8.43	7.46	***						25.64	14.53	11.82	10.62	***					
Taking medicine		40.24	18.52	15.66	17.91	***															
Getting around indoors		18.9	5.99	7.23	2.99	***															
Getting up and down the stairs		58.92	42.33	34.15	22.39	***															
Getting out of the house		71.83	55.76	44.58	38.81	***															
Shopping routine		87.2	72.77	69.14	56.92	***						90.26	78.26	70.91	69.72	***					
Housework		87.04	73.61	59.76	54.69	***											91.2	69.12	70.07	76.03	***
Paperwork or paying bills		61.6	45.33	43.21	40	***															
**ASCOT Domains**		**PERSONAL SAFETY without LTC services**					**SOCIAL PARTICIPATION without LTC services**					**OCCUPATION without LTC services**									
	**Response Levels**	**High Needs**	**Some Needs**	**No Needs**	**Ideal State**	**F stat Sign.**	**High Needs**	**Some Needs**	**No Needs**	**Ideal State**	**F stat Sign.**	**High Needs**	**Some Needs**	**No Needs**	**Ideal State**	**F stat Sign.**					
	**n**	**121**	**164**	**164**	**133**		**114**	**167**	**149**	**166**		**86**	**166**	**145**	**175**						
(I)ADL limitations index, mean (sd)		18.27 (7.60)	14.63 (7.47)	12.38 (7.10)	10.96 (8.34)	22.65***	15.69 (8.10)	14.56 (7.51)	14.26 (8.73)	11.49 (7.40)	7.18***	18.22 (8.31)	15.11 (7.78)	12.52 (7.62)	11.62 (7.40)	16.99***					
Getting in and out of bed	% can only do with help	35.11	17.34	14.53	11.97	***						38.54	21.14	15.38	12.97	***					
Washing hands and face												20.83	10.29	7.69	4.86	***					
Taking a bath or shower		80.00	67.63	60.47	56.34	***						77.08	76.00	60.00	56.76	***					
Dressing or undressing												57.29	44.57	29.49	31.35	***					
Using the toilet												34.74	20.00	11.54	10.81	***					
Eating, including cutting up food												26.04	16.00	14.10	9.73	***					
Taking medicine		34.62	35.84	18.24	19.15	***						32.29	35.43	20.78	21.20	***					
Getting around indoors		19.85	10.98	6.40	5.63	***						29.17	12.57	7.05	4.86	***					
Getting up and down the stairs		61.11	50.29	38.82	34.31	***						65.56	50.58	38.82	36.81	***					
Getting out of the house		74.81	60.82	56.40	45.77	***						74.74	63.79	52.56	51.89	***					
Shopping routine		93.8	79.41	71.60	64.03	***						92.63	79.19	71.71	70.00	***					
Housework		88.37	79.53	71.43	64.03	***						90.43	80.35	69.54	66.48	***					
Paperwork or paying bills												61.29	55.75	47.06	43.89	***					

Notes: ANOVA ((I)ADL limitations index) and Fisher’s exact test (individual (I)ADLs) were used, ^a^ highest two levels of attribute are collapsed

***significant at 1% level, ** significant at 5% level, * significant at 10% level; Benjamini & Hochberg correction for multiple testing applied

Source: WU, EXCELC INT SU AUT 2016/2017

#### Control over daily life

As anticipated, the CASP-12 subscale score, a measure for older people’s control and autonomy, was related to *Control over daily life* for the *current* QoL state (h3) at the 1% level (persons with higher levels of control & autonomy reported higher *Control over daily life*) (Table 8). Selected EQ-5D items (self-care and usual activities) (h-cont1), (I)ADL impairment index (h4), home design (h-cont3) and local access (h-cont4) were also significantly associated with current ASCOT *Control over daily life* in the expected directions. Problems with the EQ-5D items self-care and usual activities and (I)ADL limitations were associated with lower outcomes in this domain; home design meeting the service user’s needs and local area accessibility were associated with higher outcomes (Table 9). As measures of need, (I)ADLs were expected to reflect situations without support from service providers. All (I)ADLs were significantly associated with the *expected* QoL state in the domain *Control over daily life* (h-cont2). Home care service users with higher (I)ADL impairments were more likely to report low levels of *Control over daily life* if they had no care services (expected QoL-state) (h4) (Table 10).

#### Personal cleanliness and comfort

As hypothesized, the EQ-5D item ‘self-care’ (a person’s ability to wash and dress) was related to the *current Personal cleanliness and comfort (h-clean1)* item, people with no problems with self-care were more likely to report better states (Table 9). Home design (h-clean3) was also significantly associated with *Personal cleanliness and comfort*, meaning that service users reporting to have a home that meets their needs had higher states in the current ASCOT *Personal cleanliness and comfort* domain on average (Table 9). Concerning the *expected* ASCOT *Personal cleanliness and comfort* state in absence of service provision, we found the personal care ADLs (washing hands and face, taking a bath, (un)dressing and using the toilet) to be significantly associated with this ASCOT domain (Table 10), with those needing help with these ADLs being more likely to report higher needs in the *expected* QoL-state without care services in the ASCOT *Personal cleanliness and comfort* domain (h-clean2).

#### Food and drink

The CASP-12’s autonomy and control subscore was found to be strongly associated with the ASCOT *Food and drink* attribute for *current* states with care services (h3) (Table 7). Food-related (I)ADLs (eating and shopping) were highly significantly associated with the *expected* state (Table 8), as was the ADL ‘using the toilet’ (h-food1).

#### Accommodation cleanliness and comfort

In line with the hypotheses, a positive association with the interviewer’s assessment of cleanliness of the respondent’s home (h-acco3) and with home design (h-acco2) was observed for the *current* QoL-state of the ASCOT domain *Accommodation cleanliness and comfort* (Table 9). Furthermore, the ability to do housework (IADL) was negatively related with the expected QoL state for *Accommodation*, i.e. in the absence of LTC services (h-acco1) (Table 10).

#### Personal safety

For the *Personal safety* domain, the variables hypothesized to be related, namely all EQ-5D items (h-safe1), home design (h-safe4), local access (h-safe5) and the CASP-12 autonomy and control subscore (h3), were significantly associated with this ASCOT domain for current states, i.e. with care services (Tables 8 and 9). People with no problems in the EQ-5D-items, a higher mean CASP-12 autonomy and control subscore, a home that meets (most of) their needs and who can get around locally are more likely to report higher states. For the *expected* ASCOT state for this domain, we found significant associations with all of the (I)ADLs investigated (‘getting in and out of bed’, ‘taking a bath/shower’, ‘taking medicine’, ‘getting around indoors’, ‘getting up and down the stairs’, ‘getting out of the house’, ‘shopping’ and ‘housework’) (h-safe2) (Table 10).

#### Social participation and involvement

We expected social contact variables (indicating the number of social interactions a person had) to be closely related to the *Social participation and involvement* domain (h-soci2). All three social contact variables in Table 9 were significantly associated with the *current* QoL state for the *Social participation and involvement* domain. Those who had weekly contact with friends/relatives or neighbors were more likely to report better states in this ASCOT domain. Furthermore, a significant association between local access and this ASCOT domain (h-soci1) was found (Table 9). None of the individual (I)ADLs seemed suited for validation of the *expected* state of the *Social participation and involvement* domain, as they do not capture social activities or even clear prerequisites for social participation. We did, however, find a significant association between overall (I)ADL limitations and the expected QoL-state for this ASCOT domain (h4) (Table 10).

#### Occupation

The EQ-5D item ‘usual activities’ was used to validate the *current* ASCOT domain *Occupation* (h-occu1)*.* By asking whether people spend time with things they enjoy, the ASCOT domain reflects a broader concept than EQ-5D, which assesses whether respondents are able to perform their usual activities (irrespectively of their wishes to do so). Nevertheless, there is considerable conceptual overlap, and the items were significantly related at the 1% level, with people having problems with their usual activities being more likely to report lower levels of ASCOT *Occupation* (Table 9). In line with our hypotheses, *current* ASCOT *Occupation* was significantly positively associated with the CASP-12 subscale (h3) (Table 8) and local accessibility (h-occu3) (Table 9). For the *expected* state, we checked for associations with all (I)ADL items (as functional impairments in any areas are expected to limit an individual’s ability to spend their time as they wish) and found significant associations with all of them (h-occu2) (Table 10).

#### Dignity

In the *Dignity* domain, respondents are asked to assess how the way they are helped affects their self-esteem. ASCOT contains another question related to dignity (asking how *having* help affects one’s self-esteem), but this item only serves as a filter and is not used in the final score calculation. Therefore, we focus on the aforementioned item for validation purposes. As expected, both satisfaction with care services (h-dig2) as well as the overall process quality score (containing variables relating to organization and interpersonal aspects of service provision) (h-dig1) were highly significantly related with the ASCOT *Dignity* domain (Table 11). As hypothesized, the CASP-12 autonomy and control subscore (h3) was significantly associated with the *Dignity* domain (Table 8).

**Table 11:** Relationship between the German version of the ASCOT Dignity domain and related variables

		DIGNITY ('the way I’m helped')			
Care process characteristics	Response levels	High/Some Needs	No Needs	Ideal State	F stat Sign.
	**n**	**57**	**143**	**424**	
Service quality index	mean (sd)	24.26 (5.08)	26.50 (4.09)	28.00 (3.79)	19.10 ***
Satisfaction with care services	% extremely satisfied	35.09	59.44	85.11	***

Notes: ***significant at 1% level, Benjamini & Hochberg correction for multiple testing applied

Source: WU, EXCELC INT SU AUT 2016/2017

## Discussion

In this study, we reported on the cross-cultural adaptation of the German ASCOT instrument for LTC service users (INT4 version) and provided evidence on its linguistic, content and construct validity using two data sources. Cognitive interviews gave insight into the overall understanding of the instrument and helped to adjust the wording and interviewer prompts in the final version of the German ASCOT instrument for service users. In addition, they provided good evidence to support linguistic as well as content validity of the German translation of ASCOT for service users.

Although forward and back translations are well established in guidelines for standardized translation processes, we found a focus on conceptual equality (instead of strict literal translations) helpful as a guiding principle. The concept clarification guide provided by the translation company in consultation with the English ASCOT development team supported the translation and adaptation; some challenges, however, remained. The way of expressing some living situations and QoL-states seemed to differ between English-speaking and German-speaking participants, which posed challenges for the translation into German, e.g. *Control over daily life* and *Dignity*. Other domains were well understood although the German translation of ASCOT needed more and longer words than the English original to express the same content, which is possibly due to German being wordier. Overall, the cognitive interviews suggested that the Austrian home care service users understood the ASCOT items and response options sufficiently well. For face-to-face interviews using ASCOT for service users, we recommend using showcards as a visual aid for questions and response categories as these can make it easier for the respondents to retain lengthy phrases, to recall response options and thus avoid the necessity of interviewers repeating questions.

Significant associations between the overall ASCOT scores (*current* and *expected*) and related scales and subgroups of service users were found for the German instrument. As (I)ADLs and EQ-5D capture functioning of the care recipient, we found a higher correlation with the expected ASCOT score, which measures the QoL situation in the absence of LTC services. In line with the findings of previous studies [5, 21, 31, 40], the analyses of survey data provided strong evidence to support the construct validity of the German adaptation of the ASCOT domains *Control over daily life*, *Personal cleanliness and comfort*, *Accommodation* and *Social participation and involvement* for the current LTC-QoL states. As reported by Malley et al. [31] referring to the unique characteristics of ASCOT as a measure for care service effects on older people’s QoL, the lack of comparative outcome measures, however, challenged the assessment of convergent validity, which was particularly the case for the LTC-QoL states in the absence of services.

The analyses presented here also broadly support the construct validity of German adaptation for the ASCOT domains *Occupation*, *Personal safety* and *Dignity* items. The service quality index was significantly associated with *Dignity*, this is in line with van Leeuwen [21]. In contrast to Malley et al. [31], we found a significant relationship between the German translation of the *Dignity* item and the CASP-12 subscale on control and autonomy. For the German adaptation of the *Personal safety* domain*,* we found significant associations with variables related to well-being and health as well as the respondents’ environment. Service users with lower health and well-being felt less safe on average, as did those whose environment was not designed to meet their needs. This could have implications for the provision of LTC services and the aim to help people feel safe [31].

The *Food and drink* domain was not easy to validate due to a skewed distribution of answers related to the current LTC-QoL state of this ASCOT domain and the lack of related constructs and variables, with the exception of the CASP-12 subscale. Additionally, we found a strong relationship between the food-related ADLs and the QoL-states in the absence of services (expected ASCOT *Food and drink* domain) which confirms that the variables assessing limitations in coping with daily life correspond with the QoL-state without LTC services (‘expected LTC-QoL’).

There are some limitations associated with this study: first, the study sample only includes older people receiving care services at their homes. Thus, the presented conclusions concerning the construct validity of ASCOT hold only for this group. The sample includes mostly LTC service users who were born in Austria (89%) and/or were eligible for the LTC allowance, a universal cash benefit, granted on the need of long-term care, irrespective of income (92%). While the distribution of age, sex and LTC allowance levels in the survey sample is representative for the Austrian target population, results might not be generalizable to other service user groups and LTC settings. Second, ASCOT for service users consists of two instruments that differ in their administration mode (INT4 – interview version; SCT4 – self-completion version) and the scope of data collection. Both instruments have the same wording for assessing the current LTC-QoL state, but only the INT4 version, evaluated in this paper, also includes the LTC-QoL in absence of services to calculate LTC-QoL effects. It seems safe to assume that the results for the linguistic, content and construct validity hold true for current LTC-QoL in both instrument types, but further evidence on the properties of the cross-culturally adapted SCT4 is recommended. Third, as a result of the limited budget of the project, the assessment of the German translation of ASCOT solely used cross-sectional data which do not allow to explore measurement properties that require measurements for more than one point in time, such as test-retest reliability or responsiveness. Future work could help to gain more insight into the measurement properties of the German version of the instrument, such as feasibility, test-retest reliability, or responsiveness.

As considerable experiences with cross-cultural adaptation and evaluation of properties of the ASCOT measures are available to date [6, 7, 21], future research may consolidate all available findings and suggest a Minimum Data Set (MDS) as well as reference values from previous assessments of measurement properties. This may facilitate assessment processes and assure quality of future evaluation of translated ASCOT measures while giving leeway for future research to apply new or other methods to gain insight into the performance of cross-culturally adapted instruments for assessing the effects of LTC service provision on service users’ QoL.

## Conclusions

We found good evidence for a valid cross-cultural adaptation of the German version of ASCOT for service users. Both qualitative and quantitative data sources turned out useful to show that the items were well understood and the translations appropriate. The measures were sufficiently associated with conceptually-related constructs and the translated instrument was able to differentiate between service user groups. The German version of the ASCOT service user instrument is well-suited for assessing LTC-related quality-of-life outcomes in German-speaking countries and may also be used for comparative research, since ASCOT is available in several languages. In addition, the variables used for construct validation may serve as a basis for the establishment of a Minimum Data Set (MDS) of variables needed to validate translated versions of the ASCOT service user instrument. Further research on the reliability and feasibility of the German ASCOT in different care settings is encouraged.

## Data Availability

The datasets generated and analysed during the current study are not publicly available, as public use and data sharing are not covered by the informed consent.

## Abbreviations

ASCOT: Adult Social Care Outcomes Toolkit
COSMIN: COnsensus-based Standards for the selection of health Measurement INstruments
EXCELC: EXploring Comparative Effectiveness and efficiency in Long-term Care (research project)
AUT: Austria
CAPI: Computer-assisted personal interview
CASP-12 control and autonomy subscale: Six-item subscale taken from the Control, Autonomy, Self-realization and Pleasure measure (CASP-12) focusing on the control and autonomy dimensions
EQ-5D: EuroQol five-dimensional questionnaire for assessing HRQoL
EQ-5D-3L: Three-level version of the EuroQol five-dimensional questionnaire for assessing HRQoL
FSW: Vienna Social Fund
FWF: Austrian Science Fund
HRQoL: Health-related quality of life
ICECAP-O: ICEpop CAPability measure for Older people
(I)ADL: (Instrumental) activities of daily living
INT4: Four response-level interview version of the Adult Social Care Outcomes Toolkit
LTC: Long-term care
LTC-QoL: Long-term care related quality of life
MDS: Minimum Data Set
NORFACE: New Opportunities for Research Funding Agency Cooperation in Europe
QoL: Quality of life
RCT: Randomized controlled trial
SCRQoL: Social care related quality of life
SCT4: Four response-level self-completion tool version of the Adult Social Care Outcomes Toolkit
SU: Service user
WU: Vienna University of Economics and Business

## ASCOT domain abbreviations

Cont: *Control over daily life*
Clean: *Personal cleanliness and comfort*
Safe: *Personal safety*
Occu: *Occupation*
Food: *Food and drink*
Acco: *Accommodation cleanliness and comfort*
Soci: *Social participation and involvement*
Dig: *Dignity*

## Footnotes

1 https://www.qualtrics.com/de/
